# Examining the Quasi-Steady Airflow Assumption in Irregular Vocal Fold Vibration

**DOI:** 10.3390/app132312691

**Published:** 2023-11-27

**Authors:** Xiaojian Wang, Xudong Zheng, Ingo R. Titze, Anil Palaparthi, Qian Xue

**Affiliations:** 1 Department of Mechanical Engineering, University of Maine, Orono, ME 04473, USA; 2 Department of Mechanical Engineering, Rochester Institute of Technology, Rochester, NY 14623, USA; 3 Utah Center for Vocology, The University of Utah, Salt Lake City, UT 84112, USA

**Keywords:** quasi-steady assumption, flow unsteadiness, vocal fold vibration, voice generation

## Abstract

The quasi-steady flow assumption (QSFA) is commonly used in the field of biomechanics of phonation. It approximates time-varying glottal flow with steady flow solutions based on frozen glottal shapes, ignoring unsteady flow behaviors and vocal fold motion. This study examined the limitations of QSFA in human phonation using numerical methods by considering factors of phonation frequency, air inertance in the vocal tract, and irregular glottal shapes. Two sets of irregular glottal shapes were examined through dynamic, pseudo-static, and quasi-steady simulations. The differences between dynamic and quasi-steady/pseudo-static simulations were measured for glottal flow rate, glottal wall pressure, and sound spectrum to evaluate the validity of QSFA. The results show that errors in glottal flow rate and wall pressure predicted by QSFA were small at 100 Hz but significant at 500 Hz due to growing flow unsteadiness. Air inertia in the vocal tract worsened predictions when interacting with unsteady glottal flow. Flow unsteadiness also influenced the harmonic energy ratio, which is perceptually important. The effects of glottal shape and glottal wall motion on the validity of QSFA were found to be insignificant.

## 1. Introduction

The quasi-steady flow assumption (QSFA) is a commonly used approximation in the field of biomechanics of phonation. It involves approximating time-varying glottal flow with a series of steady flow solutions based on their ‘time-frozen’ glottal shapes while ignoring unsteady flow behaviors and vocal fold motion. The assumption was originally justified based on two observations of typical human phonation [1]. Firstly, the dimensions of the glottis, length (L) and thickness (T), are small compared to the “wavelength” defined as the glottal particle velocity $\left(v_{g}\right)$ divided by fundamental frequency $\left(f_{0}\right)$. This leads to the nondimensional inequality condition of $S_{t}\ll 1$, where $S_{t}$ is the Strouhal number defined as $S_{t}=f_{0}L/v_{g}$. By adopting $f_{0},L$, and $v_{g}$ as the characteristic frequency, length, and velocity, respectively, in the process of nondimensionalization, the unsteady flow term in the Navier–Stokes equation becomes $S_{t}\frac{\partial \vec{V}}{\partial t}$, which can be dropped out from the equation through order analysis. Secondly, the velocity of glottal airflow, $v_{g}$, is much greater than the oscillation speed of the vocal fold, $v_{v}$. This yields the second nondimensional inequality condition of $V_{r}\gg D$, where $V_{r}=v_{g}/v_{v}$ is the reduced velocity, and $D=\xi_{o}/L$ is the displacement number. Here, $\xi_{o}$ is the vocal fold vibration amplitude defined as $\xi_{o}=v_{v}/f_{o}$. Through nondimensional order analysis on the boundary conditions, this inequality condition enables the static glottal wall approximation.

The QSFA approximation is a valuable tool for understanding the mechanics of human phonation and has been used in combination with other models to investigate flow-structure interactions during phonation. One example is the myoelastic–aerodynamic (MEAD) theory of phonation, which employs the Bernoulli law to explain the energy transfer mechanism during phonation [2,3]. To explore flow–pressure relationships during vocal fold vibration, researchers have conducted experiments on static glottal configurations [4,5]. In addition, computer vocal fold models such as the two-mass model [1], the three-mass model [6], and continuum models [7,8] are often coupled with steady flow solutions to model flow–structure interactions during phonation.

Despite its usefulness, the QSFA approximation has limitations. Mongeau et al. [9] investigated the dynamic characteristics of an open jet flow through a modulated convergent orifice experimentally, and the work was extended by Zhang et al. [10] by considering confined jet flows and different orifice shapes (convergent, straight, and divergent). They compared the flow measurements, such as the pressure, velocity, and flow rate of the pulsating jet, with those predicted by a quasi-steady flow model based on the Bernoulli equation. Their results showed that while the QSFA model produced accurate predictions for most of the vibration cycle, it deviated significantly during flow initiation and shutoff. In the work of Pelorson [11], unsteady pulsatile flow through a rigid uniform constriction was produced using a collapsible tube. The pressure–flow relationship of the unsteady flow was compared against the predictions of the steady Bernoulli formula and the steady Bernoulli solution accounting for a Poiseuille term. It was found that the steady flow models are not accurate during flow onset or vocal fold closure. To solve the flow more accurately and easily, Vilain et al. [12] proposed another quasi-steady flow model based on Thwaites’ method. By comparing the glottal pressure predicted by the model with the unsteady measurements on straight and rounded rigid replicas of the vocal folds, they reported that the QSFA does not stand when the glottis is almost closed. To address this limitation, Krane and Wei [13] conducted a theoretical study to assess the order of magnitude of the unsteady and convective acceleration terms in the momentum equation, which relates the transglottal pressure to the volume flow. The effects of glottal wall motion, friction, and the movement of the separation point were considered in the equation. They showed that glottal flow dynamics could be divided into an unsteady-effect-dominated flow-initiation/shutoff interval and a convective-acceleration-dominated quasi-steady interval, based on the relative importance of each term. Subsequently, Krane et al. [14] combined the theoretical work with experimentally measured flow velocity data and calculated the waveshapes of unsteady and convective accelerations. Flow velocity measurements were conducted in a scaled-up vocal fold model using digital particle image velocimetry (DPIV), and unsteady and convective acceleration terms were calculated along the centerline of the flow between the vocal folds. Their results indicated that although glottal jet inertia was nearly zero during the middle 40% of the open phase, unsteady acceleration still dominated the flow for the remainder of the cycle.

A summary of previous studies on the QSFA is displayed in Table 1. Due to the challenges of experimentally implementing the complex geometry of the vocal folds, almost all studies used simplified geometries. However, under pathological vocal fold vibrations, the glottal shape can be asymmetric and involve longitudinal wave propagations [15,16]. Since different glottal shapes can generate significantly different glottal flow dynamics [5,17], there remains a question as to whether the previous conclusions still stand when the complex geometry of the vocal folds is considered. In addition, the frequency investigated by the preceding studies concentrates in the range of 10–160 Hz. The nondimensional inequality condition of $S_{t}\ll 1$ suggests that the validity of QSFA is frequency dependent. The $S_{t}$ for glottal flow in normal speech phonation is approximately 10^−2^, estimated based on a frequency range of 100–200 Hz. However, higher vibration frequencies, such as those encountered in singing, can lead to larger $S_{t}$, which, coupled with an increase in the oscillation speed of vocal folds, may undermine the justification of QSFA. Therefore, the dependence of the validity of QSFA on frequency also needs to be explored. Another factor that affects the validity of QSFA is the air inertia effect in the vocal tract. Kucinschi and Scherer [18] showed that the inertance of air in the trachea and vocal tract generates a dynamic pressure at the glottal inlet and outlet, which interacts with the unsteady glottal flow and can cause the flow rate and transglottal pressure to vary with frequency. Thus, considering the air inertia effects in the validation of QSFA may yield different results.

The purpose of this study is to utilize numerical methods to explore the limitations of QSFA in human phonation, taking into account factors such as phonation frequency, air inertance in the vocal tract, and irregular glottal shapes. To isolate the effects of flow unsteadiness and vocal fold vibration, three simulation types were conducted. The first simulation type, dynamic simulation, involved solving the unsteady Navier–Stokes equations with prescribed vocal fold motion, including glottal wall shapes and velocity at each time step. This simulation type considered both unsteady flow and vocal fold motion effects. The second simulation type, pseudo-static simulation, computed the steady solution of the Navier–Stokes equations for each glottal wall shape with the corresponding glottal wall velocity. This simulation type considered the effect of glottal wall motion but not the effects of unsteady flow. The third simulation type, quasi-steady simulation, computed the steady solution of the Navier–Stokes equations for each glottal wall shape without incorporating the glottal wall velocity. This simulation type considered neither unsteady flow nor glottal wall motion effects. Two different vibration frequencies, 100 and 500 Hz, were simulated to test the dependence of QSFA on frequency. The effect of air inertia in the supraglottic vocal tract was tested by implementing simulations with and without a vocal tract. To address the effect of glottal shapes on QSFA, two sets of asymmetric glottal shapes that incorporate different vertical and longitudinal wave patterns on the right and left vocal folds were used. The validity of QSFA was evaluated by calculating the differences in glottal flow rate and glottal wall pressure among the dynamic, pseudo-static, and quasi-steady simulations. Additionally, a momentum budget analysis was performed to determine the magnitude of unsteady acceleration in glottal airflow and its impact on the quantified flow and wall pressure. The influence of flow unsteadiness on sound generation and perception was also investigated in this study.

## 2. Methods

### 2.1. Glottal Shapes and Movement

Two sets of time-dependent glottal shapes were generated using the surface-wave approach proposed by Smith and Titze [24]. This approach utilizes a combination of (m,n) modes to describe the wave-like motion of the vocal fold surface, where m and n represent the number of half wavelengths along the anterior–posterior and inferior–superior directions, respectively. In normal phonation, the dominant modes are the (1,0) and (1,1) modes, representing the medial–lateral and convergent–divergent motions, respectively. Higher mode numbers correspond to more complex wave motions, which can occur in voice disorders. For instance, a (2,1) mode corresponds to two half-wavelengths in the anterior–posterior direction and one half-wavelength in the inferior–superior direction. In this study, we generated two sets of asymmetric glottal wall dynamics by combining the (1,0) mode (left) and (2,1) mode (right) in the first set and the (1,1) mode (left) and (2,0) mode (right) in the second set.

**Figure 1.**
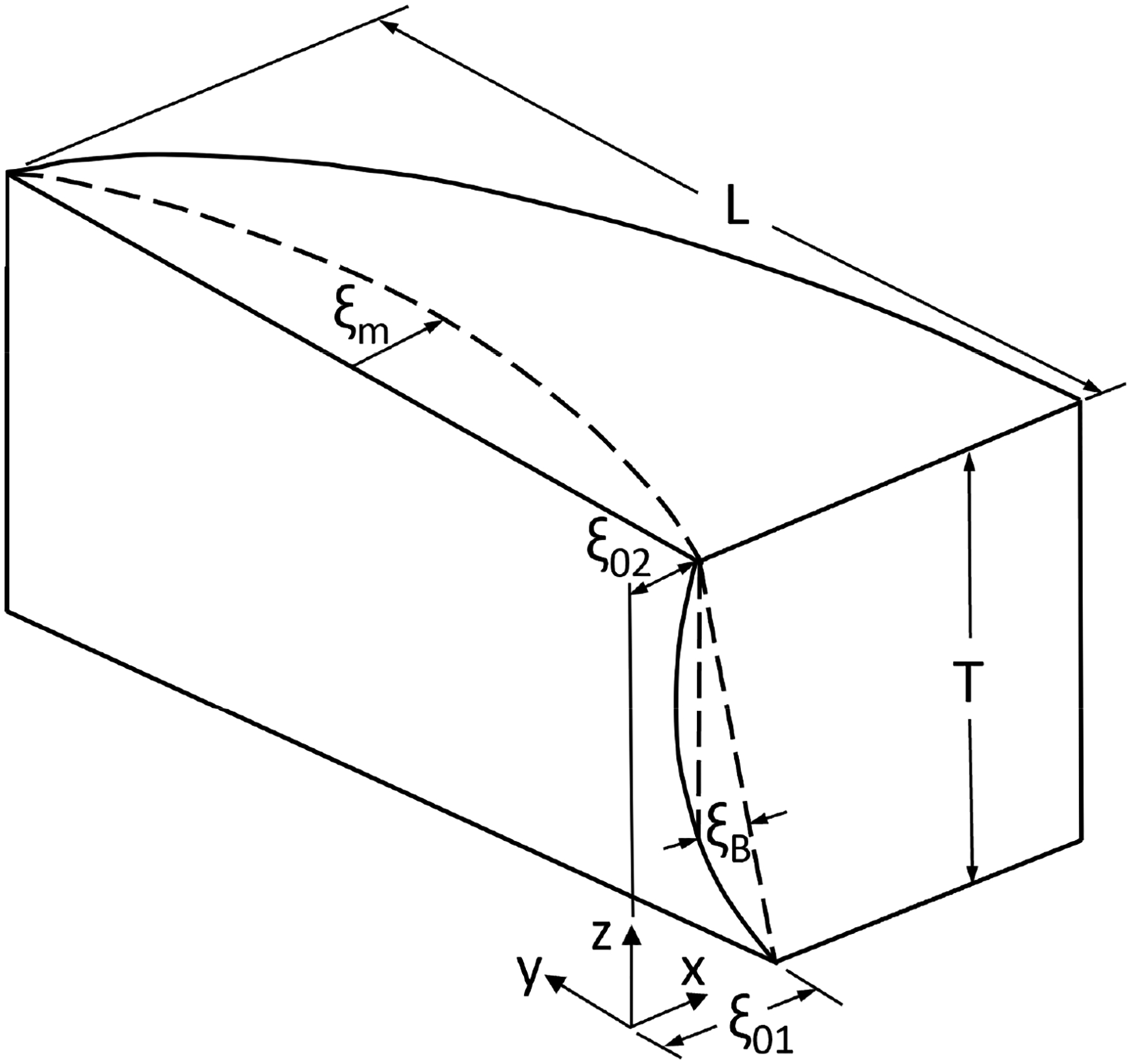
Diagram of the pre-phonatory configuration of the right medial surface.

The equations for generating the vocal fold dynamics are briefly introduced below. Taking the right vocal fold as an example, its pre-phonatory medial surface (Figure 1) was defined by a glottal half-width $\xi_{0\text{R}}$ as a function of anterior–posterior (y) and inferior–superior (z) directions:

(1) $$\xi_{0\text{R}}(\text{y},\text{z})=(1-\text{y}/\text{L})\left[\xi_{0\text{R}2}+\left(\xi_{0\text{R}1}-\xi_{0\text{R}2}-4\xi_{\text{BR}}\;\text{z}/\text{T}\right)(1-\text{z}/\text{T})\right],$$

where L and T are, respectively, the length and thickness of the vocal fold, $\xi_{0\text{R1}}$ and $\xi_{0\text{R2}}$ are, respectively, the inferior and superior glottal half-widths at the vocal process, and $\xi_{\text{BR}}$ is a surface bulging parameter, which controls the vertical curvature of the medial surface. The subscript R denotes the right medial surface. The same equation with a subscript L exists for the pre-phonatory glottal width of the left medial surface $\left(\xi_{0L}\right)$.

For the right medial surface, the modal displacement $\xi_{R}$ at any instant of time (t) is defined as follows:

(2) $$\xi_{\text{R}}\left(\text{y},\text{z},\text{t}\right)=\xi_{\text{mR}}\;\text{sin}(\text{m}\pi \text{y}/\text{L})\;\left[\text{sin}\omega \text{t}-\text{n}(\omega /\text{c})\left(\text{z}-{\text{z}}_{\text{mR}}\right)\text{cos}\omega \text{t}\right],$$

where $\xi_{mR}$ is the modal displacement amplitude, ω is the angular frequency, c is the mucosal wave speed, and $z_{mR}$ is the inflection point for the vertical half wavelength. An equivalent equation exists for the modal displacement of the left medial surface $\left(\xi_{L}\right)$. The overall three-dimensional glottal shape at any moment of time was obtained by superimposing the modal displacements on the pre-phonatory shape of the medial surfaces:

(3) $$\text{g}(\text{y},\text{z},\text{t})=\xi_{0\text{R}}(\text{y},\text{z})+\xi_{0\text{L}}(\text{y},\text{z})+\xi_{\text{R}}(\text{y},\text{z},\text{t})+\xi_{\text{L}}(\text{y},\text{z},\text{t}).$$

In this study, the vocal fold thickness, T, and length, L, were adopted from Smith and Titze [24], which are 0.8 and 1.5 cm, respectively. A uniform (parallel surface), initial posturing configuration was used, and the parameter values (in cm) were as follows:

$$\xi_{0R1}=0.10,\;\xi_{0R2}=0.10,\;\xi_{BR}=0.005,\;\xi_{0L1}=0.10,\;\xi_{0L2}=0.10,\;\xi_{BL}=0.005.$$

The vibration frequency of the vocal folds, f, was chosen to be 100 Hz and 500 Hz. The angular frequency, ω, and the mucosal wave speed, c, were defined as 2πf and Tπf, respectively. For both the right and left medial surfaces, the modal displacement amplitude $\xi_{m}$ was 0.1 cm, and the inflection point $z_{m}$ was defined as $T\times \left(0.6-0.02\xi_{B}\right)$.

**Figure 2.**
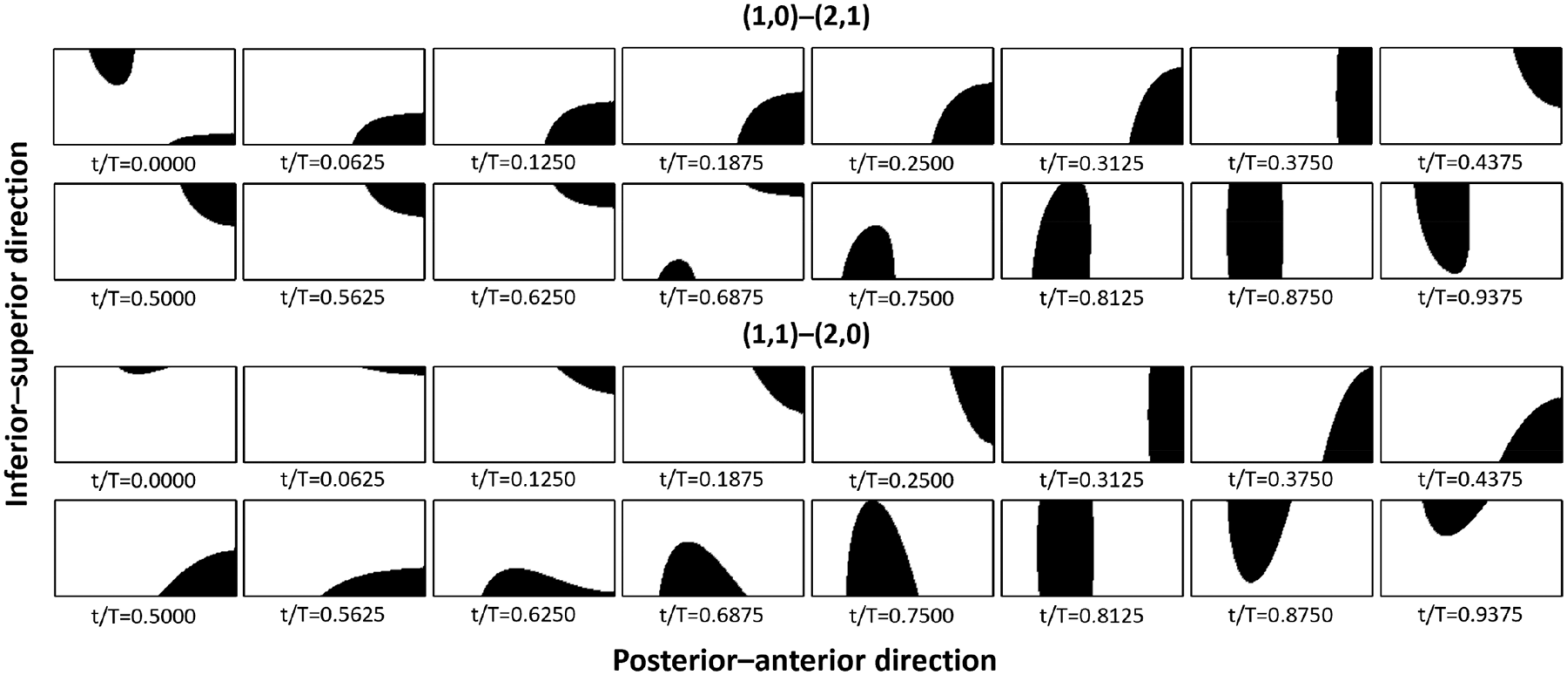
Contact patterns of the sixteen shapes of the (1,0)–(2,1) and (1,1)–(2,0) sets of glottal wall motions. For each phase, the contact area (black) and open glottis (white) are marked on the rectangular medial surface.

In this study, a total of 16 sequential static glottal shapes were extracted for each of the (1,0)–(2,1) and (1,1)–(2,0) sets of glottal wall motions. These shapes were obtained from one period of vibration (from t/T = 0 to 0.9375) at even time intervals (Δt/T=0.0625) and are shown in Figure 2. In the dynamic simulations, the position of the glottal wall at each time instant was determined through cubic spline interpolation between the 16 shapes, ensuring continuous progression from shape to shape as in normal vocal fold vibration. Unsteady flow solutions were then obtained at each time instant. For the pseudo-static simulations, each of the 16 glottal shapes was used to perform a separate numerical simulation, with the corresponding wall velocity applied on the glottal wall. This yielded steady-flow solutions for each glottal shape. In the case of quasi-steady simulations, each glottal shape also corresponded to an independent simulation but with no velocity applied on the wall. Steady-flow solutions were obtained for each of the 16 glottal shapes in this case as well.

### 2.2. Airflow Simulation

The aerodynamics of glottal airflow were numerically calculated from the 3D, unsteady, viscous, incompressible Navier–Stokes equations, which consist of the following continuity and momentum conservation equations:

(4) $$\nabla \cdot \vec{V}=0,$$

(5) $$\frac{\partial \vec{V}}{\partial t}+\left(\vec{V}\cdot \nabla \right)\vec{V}=-\frac{1}{\rho_{0}}\nabla P+v_{0}\nabla^{2}\vec{V},$$

where $\vec{V}$ is the flow velocity, P is the pressure, and $\rho_{0}$ and $v_{0}$ are flow density and kinematic viscosity, respectively. A sharp-interface immersed-boundary method was employed to solve the Navier–Stokes equations. The equations were spatially discretized and solved on nonuniform Cartesian grids using the collocated arrangements of velocity components and pressure. The second-order fractional step method of Van Kan was used to integrate the equations in time. The boundary conditions were imposed on glottal airflow by immersing the vocal fold surface, represented by unstructured triangular elements, into the Cartesian grid using a multi-dimensional ghost-cell method. More detailed descriptions of the numerical schemes are shown in Mittal et al. [25] and Zheng et al. [26].

### 2.3. Simulation Setup and Case Summary

**Figure 3.**
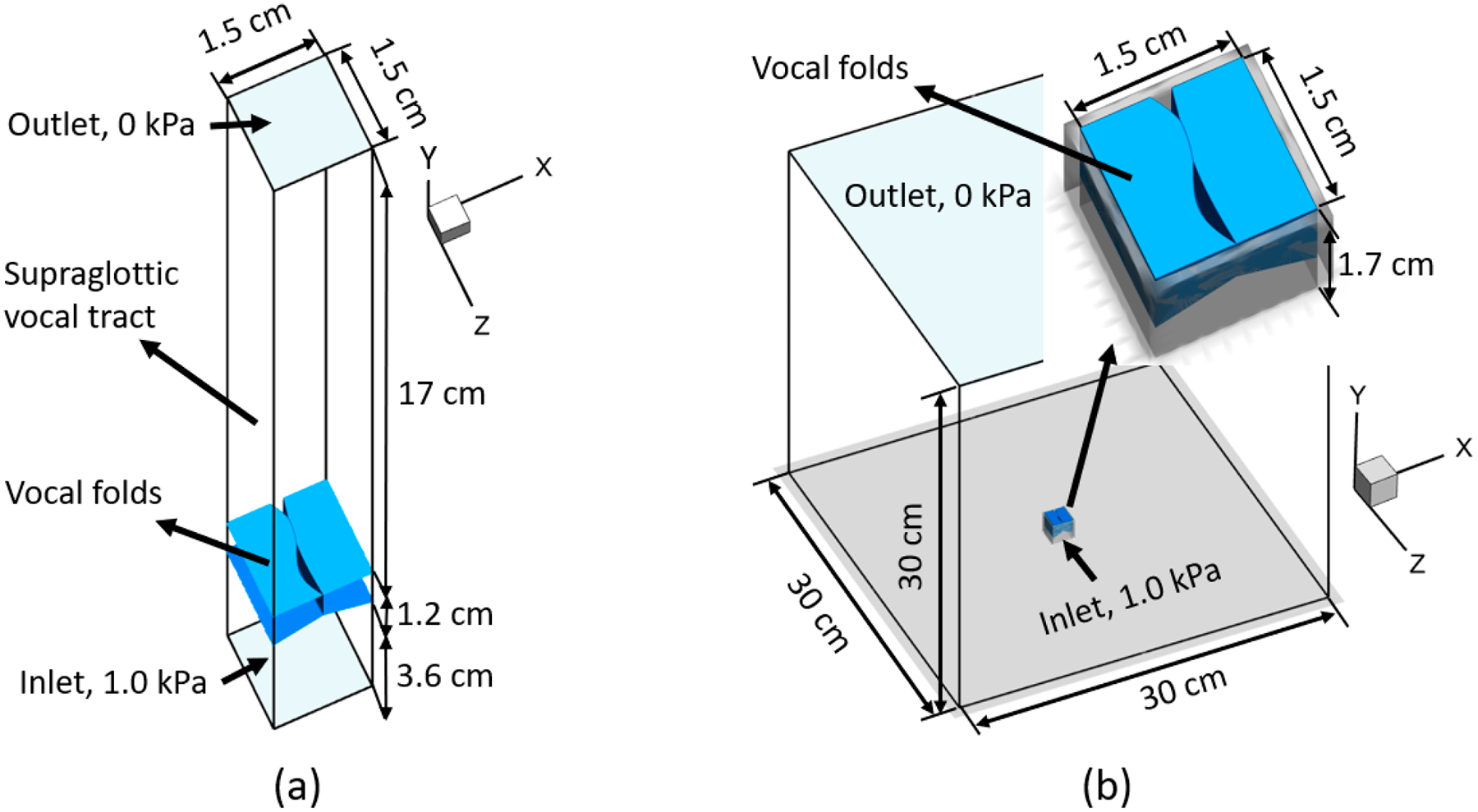
Computational domain and vocal fold model: (**a**) setup with supraglottic vocal tract. (**b**) Setup without supraglottic vocal tract. The representative glottal shape corresponds to the (1,0)–(2,1) set at t/T = 0.0000.

The computational model setup is shown in Figure 3. Figure 3a is the setup with a supraglottic tract. The vocal fold surfaces were immersed into a 1.5 cm × 21.8 cm × 1.5 cm rectangular computational domain, which consists of a 17 cm-long supraglottic vocal tract, a 3.6 cm-long trachea, and a pair of 0.8 cm-thick vocal folds. The computational domain was discretized by a 128 × 128 × 96 (x × y × z) Cartesian grid, and grid independence was achieved with this grid. Dirichlet boundary conditions for pressure of 1.0 kPa and 0 kPa were applied at the inlet and exit of the computational domain, respectively. No-slip and no-penetration boundary conditions were imposed on the trachea and vocal tract walls. Figure 3b is the setup without the supraglottic tract. The vocal folds were located at the bottom center of a cubic computational domain, 30 cm × 30 cm × 30 cm, mimicking a large open environment. A 256 × 128 × 128 (x × y × z) Cartesian grid was used to discretize the computational domain. The grid around the vocal folds was 164 × 72 × 92 (x × y × z), which provided a similar grid resolution as the setup with the supraglottic tract. Similar to the former setup, constant pressure boundary conditions were applied at the entrance of the trachea and top surface of the computational domain, 0 kPa and 0 kPa, respectively. The left, right, front, and back surfaces, as well as the bottom surface outside the trachea area, were treated as no-slip and no-penetration boundaries. For both setups, the surface of each vocal fold was discretized into 71,400 triangular elements with 0.01 cm resolution. In the dynamic simulations, a 0.006 cm minimum space between the left and right medial surface in contact area was enforced, which was necessary for the success of airflow simulation.

In the dynamic simulations, the time steps of 1.184 × 10^−6^ s and 1.016 × 10^−6^ s were used for the case of 100 Hz and 500 Hz vibration frequency, respectively. Such time steps were chosen for (1) outputting the results at the specified 16 phases and (2) complying with the Courant–Friedrichs–Lewy condition. The same time steps were used for pseudo-static and quasi-steady simulations. For a few cases that experienced numerical convergence problems, the time step was appropriately reduced. Time independence was achieved for all three types of simulations. For each dynamic simulation, glottal airflow was simulated for two cycles, reaching steady cycles based on the observation that cycle-to-cycle flow variations were very small. For each pseudo-static or quasi-steady simulation, glottal airflow was calculated until a sustained steady state was reached. The simulations were run on the XSEDE COMET cluster, which used Intel’s Xeon Processor E5–2600 v3 family. One hundred and twenty-eight processors were used for each dynamic or pseudo-static simulation, and sixty-four processors were used for each quasi-steady simulation. The computational time of the dynamic simulations ranged from 40 h to 240 h, depending on the vibration frequency and number of grids. The average computational time for a pseudo-static or quasi-steady simulation was around 20 h.

The total number of simulations included in this study was 200, with 8 dynamic, 128 pseudo-static, and 64 quasi-steady cases. For each of the two sets of glottal wall motions, setups were implemented with two different supraglottic configurations (with and without the supraglottic tract) under two different vibration frequencies (100 and 500 Hz), which adds up to the 8 dynamic cases. Based on each dynamic case, 16 pseudo-static simulations, each with a glottal wall velocity extracted from the dynamic simulation, were built, resulting in 128 pseudo-static cases. For the quasi-steady cases, since there were no velocities on the glottal wall and the simulations were frequency-independent, the number of cases was reduced to 64.

## 3. Results

### 3.1. Glottal Flow Rate Comparison

**Figure 4.**
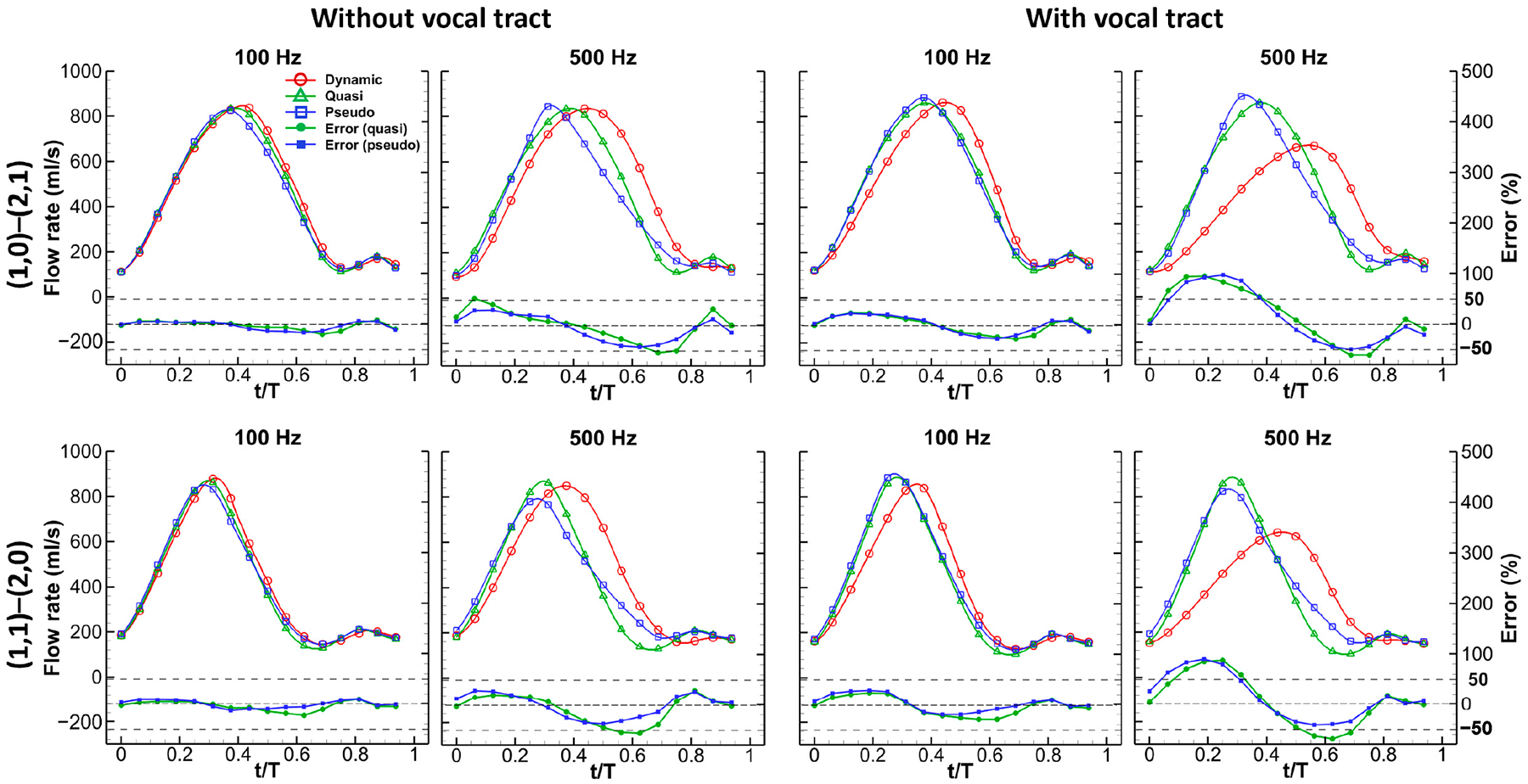
Flow rate waveforms of the three simulations and percent errors of flow rate between quasi-steady/pseudo-static and dynamic simulation over one vibration cycle.

In Figure 4, we compare the glottal flow rates over one vibration cycle across different simulation setups for each of the two sets of glottal shapes, including dynamic, pseudo-static, and quasi-steady simulations. The flow rate waveforms for the quasi-steady and pseudo-static simulations were obtained using the “spline” function in MATLAB to interpolate over the 16 discrete flow rate points. To assess the accuracy of the pseudo-static and quasi-steady assumptions, we calculated the percent errors of the flow rate by comparing and normalizing the values obtained from these simulations to those obtained from the dynamic simulations under corresponding configurations. At a frequency of 100 Hz, the flow rate waveforms of the dynamic, quasi-steady, and pseudo-static simulations for the (1,0)–(2,1) glottal shape set showed significant similarities when the vocal tract was not considered. The cycle-averaged absolute percent errors for the quasi-steady and pseudo-static simulations were 6.5% and 7.2%, respectively. The errors were primarily due to a slight phase difference during flow deceleration. However, including the vocal tract led to larger phase differences, causing the quasi-steady and pseudo-static simulations to deviate from the dynamic cases, with cycle-averaged absolute percent errors increasing to 14.7% and 15.0%, respectively.

At the higher frequency of 500 Hz, significant departures of flow rate from those of the dynamic case were observed, with cycle-averaged absolute percent errors of 24.1% and 22.2% for the quasi-steady and pseudo-static simulations, respectively, when the vocal tract was not considered. When the vocal tract was included, the errors nearly doubled, with cycle-averaged absolute percent errors of 46.1% and 44.5% for the quasi-steady and pseudo-static simulations, respectively. These data indicate that flow rate errors nearly tripled as the vibration frequency increased to 500 Hz, including the fact that the vocal tract in the simulation nearly doubled the errors. Similar observations were made for the flow rate waveforms of the (1,1)–(2,0) set, with cycle-averaged absolute percent errors ranging from 8.0% to 41.2% for the quasi-steady and pseudo-static simulations compared to the dynamic case. The errors were nearly identical for the two different sets of glottal shapes, indicating that the accuracy of flow rate calculation by quasi-steady or pseudo-static approximation was insensitive to glottal shapes.

Figure 4 also shows that the flow rate error varies during a vibration cycle for all the cases, with the error increasing and then decreasing during glottis opening and closing. The highest errors were observed in the middle of opening and closing, corresponding to peak flow acceleration and deceleration. This variation in errors was more prominent at the 500 Hz frequency. The nonuniform error levels suggest that the importance of unsteady effects varies within one vibration cycle. Furthermore, at 100 Hz frequency, the flow rate waveforms of quasi-steady and pseudo-static cases almost overlap, indicating that glottal wall motion has a negligible impact on flow rates at low vibration frequency. However, at 500 Hz frequency, discrepancies between the two flow rate waveforms are apparent, particularly during flow deceleration. Generally, the quasi-steady cases display higher cycle-averaged errors than the pseudo-static cases, indicating that incorporating glottal wall motion in the model can improve the accuracy of flow rate prediction at high vibration frequency.

### 3.2. Strouhal Number and Velocity Ratio Analysis

To investigate how the variations of flow rate errors are associated with the quasi-steady assumption, the Strouhal number of each dynamic case was calculated. In the equation $S_{t}=f_{0}L/v_{g},f_{0}$ and L are, respectively, the vibration frequency and thickness (0.8 cm) of the vocal fold. $v_{g}$ is the cycle-averaged upstream flow velocity (taken at glottis inlet) in each case. The value of $S_{t}$ for each dynamic case is listed in Table 2. At 100 Hz vibration frequency, $S_{t}$ is of the order of 0.03 for all cases, while $S_{t}$ increased nearly fivefold (approximately 0.15) at 500 Hz frequency. Note that the value of $S_{t}$ at 100 Hz frequency falls well within the range (0.01 to 0.1) accepted for QSFA [12,14], whereas the value of $S_{t}$ at 500 Hz frequency is beyond the range. Therefore, the very large errors of flow rate at 500 Hz frequency are due to the breakdown of QSFA. In other words, the unsteady effects of glottal flow play an important role at a high phonation frequency and cannot be neglected.

**Figure 5.**
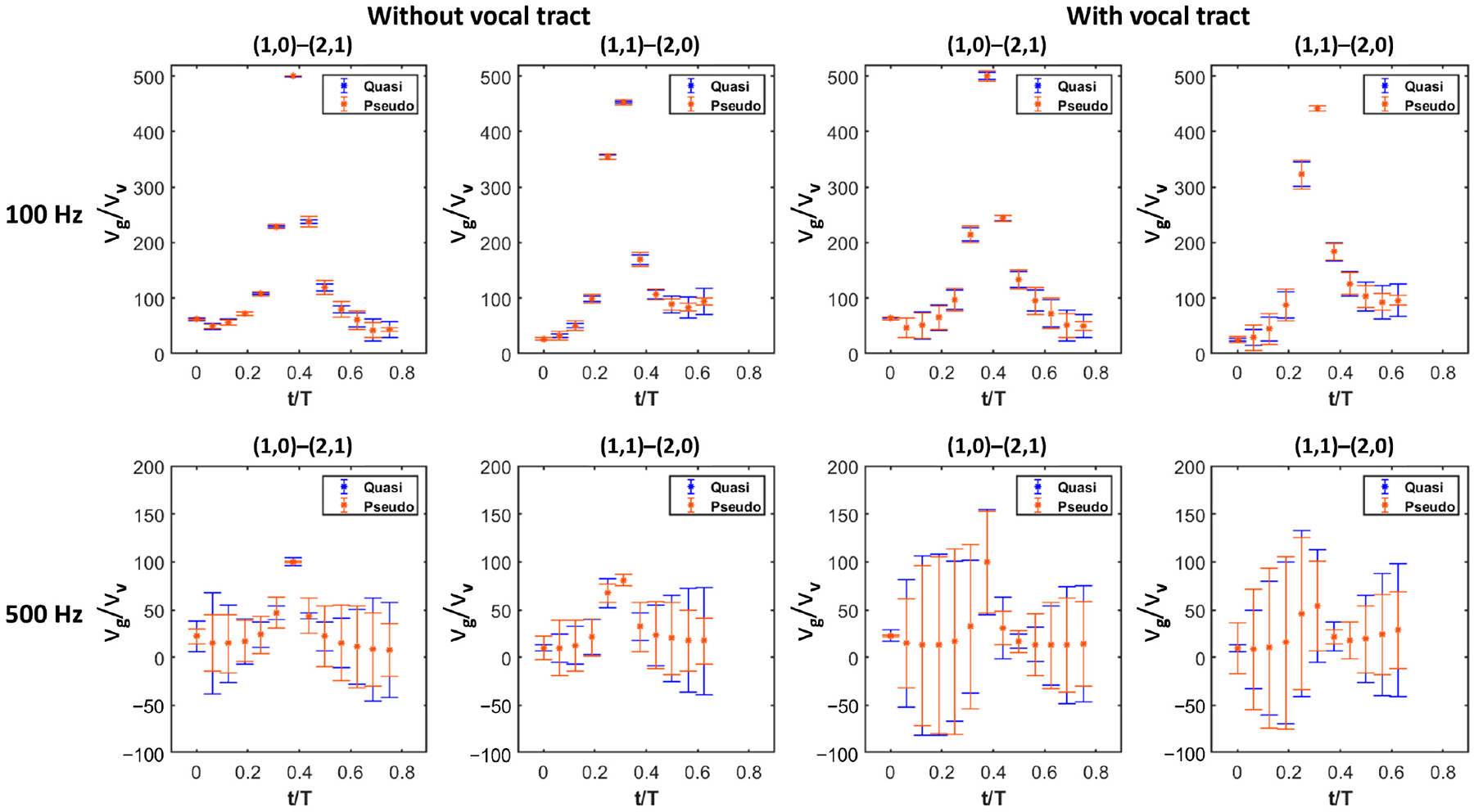
$v_{g}/v_{v}$ versus t/T during the open phase, with the flow rate errors being presented in the form of error bars. Maximum values of $v_{g}/v_{v}$ for the (1,0)–(2,1) set at 100 Hz are much larger than 500. They are limited to 500 for a better view of other $v_{g}/v_{v}$ values.

The ratio of flow velocity to vocal fold vibration velocity, $v_{g}/v_{v}$, was also calculated during each of the 16 phases for the dynamic cases. In the calculation $v_{g}/v_{v},v_{g}$ is the mean flow velocity at the glottis inlet, and $v_{v}$ is the averaged vibration velocity of the medial surface on the side of mode with one half wavelength in the longitudinal direction. Figure 5 plots $v_{g}/v_{v}$ versus t/T during the open phase for each case, and the absolute percent errors of flow rate between the quasi-steady/pseudo-static and dynamic simulations are displayed in the form of error bars at each data point (the length of error bars denote the magnitude of errors). Figure 5 shows that $v_{g}/v_{v}$ is generally in an inverse relationship with flow rate error. For t/T < 0.2 and t/T > 0.5, $v_{g}/v_{v}$ is relatively small but accompanied by a large flow rate error. While in the interval 0.2 < t/T < 0.5, $v_{g}/v_{v}$ has a high value, but the error is significantly reduced. It is observed that, in the cases with the frequency of 500 Hz and the supraglottic tract included, this inverse relationship is not as evident as in other cases, probably because the air inertia effect in the vocal tract dominates the flow rate error. Figure 5 also shows that for the same phase, $v_{g}/v_{v}$ has a much smaller value at 500 Hz than at 100 Hz, which is consistent with the above inverse relationship since flow rate errors are much larger at 500 Hz. These observations verify that a large velocity ratio between glottal flow and vocal fold vibration is necessary for the legitimacy of the quasi-steady assumption. Lastly, it is also noted that the variations of flow rate errors with $v_{g}/v_{v}$ show a similar trend for quasi-steady and pseudo-static cases.

### 3.3. Errors of Important Aerodynamics and Sound Spectrum Parameters

To gain insight into the influence of QSFA on voice outcomes, errors of several important aerodynamic and sound spectrum parameters were computed and compared between quasi-steady/pseudo-static and dynamic cases. The resulting errors are tabulated in Table 3. Peak flow errors were small (≤5.35%) for all cases at 100 Hz and 500 Hz without the vocal tract. However, significant errors were observed for the 500 Hz case with the vocal tract, where the dynamic simulation showed a marked decrease in peak flow not seen in the quasi-steady/pseudo-static simulations. This decrease in peak flow at higher frequencies is consistent with previous experimental and numerical findings by Kucinschi and Scherer [18], who attributed it to the inertial effects of the air in the trachea and vocal tract that resist changes in flow rate and grow with frequency. Mean flow errors were small (≤3.94%) at 100 Hz but more significant at 500 Hz, with a maximum error of 14.07%.

Phase shift errors, calculated as the phase difference in peak flow between the quasi-steady/pseudo-static and dynamic cases, were substantial for all cases, with negative values indicating delayed peak flow in the dynamic case. The phase shift values significantly increased when a vocal tract was considered due to air inertia in the tract that skews the flow rate waveform to the right. Additionally, as frequency increased from 100 Hz to 500 Hz, a significant increase in phase shift was observed for all cases. The pseudo-static assumption generated larger phase shift errors than the quasi-steady assumption, indicating that glottal wall motion brings the peak flow to an earlier time.

MFDR errors were relatively small for the quasi-steady case at both 100 Hz and 500 Hz without a vocal tract, while large errors were observed for the pseudo-static case, which could be attributed to larger phase shift errors. MFDR errors increased only for the quasi-steady case when a vocal tract was considered, and no strong correlation between errors and frequency was observed in Table 3.

Spectral analysis was conducted by measuring the spectral amplitude differences between the first two harmonics (H1–H2) and the first and fourth harmonics (H1–H4), respectively. The spectral slope was calculated by fitting the first 20 harmonics of each case using linear regression in MATLAB. The results showed that errors of H1–H2 and H1–H4 significantly increased with frequency in simulations without a vocal tract, while errors of spectral slope did not appear to be related to frequency. In simulations with a vocal tract, errors of H1–H2 exhibited the same frequency-dependent characteristic, while errors of H1–H4 did not. These observations suggest that although flow unsteadiness does not have a noticeable impact on sound intensity (MFDR), it still strongly influences sound quality and is, therefore, perceptually significant.

Overall, applying QSFA leads to small errors in flow rate prediction but large errors in phase shift prediction, with substantial increases in errors when a vocal tract is included or at high vibration frequencies. The pseudo-static assumption does not improve flow rate waveform prediction but actually generates larger errors in phase shift and MFDR predictions. QSFA also influences sound spectrum amplitude differences, e.g., H1–H2 and H1–H4, which can influence sound quality.

### 3.4. Errors of Glottal Pressure

**Figure 6.**
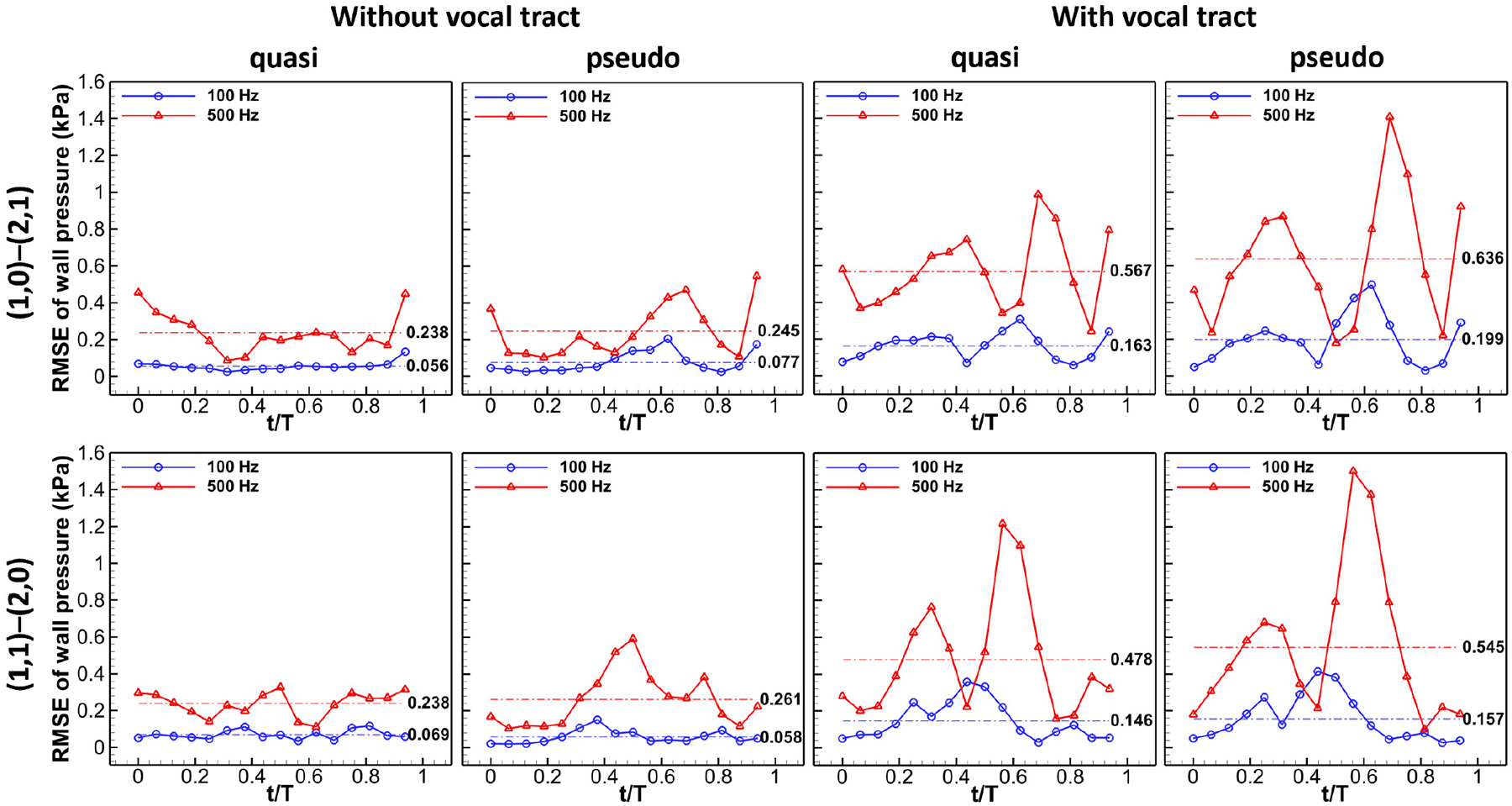
RMSE of glottal wall pressure between the quasi-steady/pseudo-static and dynamic case over one vibration cycle.

Figure 6 illustrates the root-mean-square error (RMSE) of glottal wall pressure between the quasi-steady/pseudo-static and dynamic cases over one vibration cycle. First, the pressure differences between the quasi-steady/pseudo-static and dynamic cases were calculated for the same element node on the medial surface. Then, the RMSE was obtained by taking the square root of the arithmetic mean of the squares of the pressure differences. In Figure 6, it can be observed that the RMSE of glottal wall pressure is small at 100 Hz, but it increases significantly as the frequency increases to 500 Hz. For example, the cycle-averaged RMSE ranges from 0.056 to 0.077 kPa for all cases at 100 Hz without a vocal tract, while it ranges from 0.238 to 0.261 kPa for all cases at 500 Hz without a vocal tract, representing an almost four-fold increase.

By comparing the cases with and without the vocal tract, a significant increase in the RMSE of glottal wall pressure is also observed. Figure 6 indicates that the RMSEs of glottal wall pressure are almost at the same level for the two different sets, suggesting that glottal shapes do not have a significant effect on the errors of glottal pressure. Although a more significant error is commonly observed during the flow deceleration stage for pseudo-static cases, the difference in the cycle-averaged RMSE of glottal wall pressure between the quasi-steady and pseudo-static cases is small.

Overall, the trend of errors of glottal pressure is consistent with the observations made above, that errors are small at low frequencies without a vocal tract. As the vibration frequency increases or a vocal tract is included, the errors of glottal pressure increase remarkably.

### 3.5. Momentum Budget Analysis

A comprehensive momentum budget analysis was conducted for each case to evaluate the relative importance of unsteady acceleration in glottal flow. This approach facilitates the comparison of each term, namely the unsteady term, convective term, pressure term, and shear term in the Navier–Stokes momentum equation, along with their variations throughout the cycle. The analysis aims to provide information on the dynamic interplay of these terms, shed light on their respective contributions to glottal flow dynamics, and reveal important instances where the unsteady term plays an important role.

The Navier–Stokes momentum equation was nondimensionalized as follows:

(6) $$\frac{\partial \vec{V}}{\partial t}+\left(\vec{V}\cdot \vec{\nabla }\right)\vec{V}=-\vec{\nabla }P+\frac{1}{Re}{\vec{\nabla }}^{2}\vec{V},$$

where $\frac{\partial \vec{V}}{\partial t}$ is the unsteady acceleration term, $(\vec{V}\cdot \vec{\nabla })\vec{V}$ is the convection term, and $\vec{\nabla }P$ and $\frac{1}{Re}{\vec{\nabla }}^{2}\vec{V}$ are the pressure and shear stress terms, respectively. To quantify and compare the magnitude of different terms, the glottis was taken as the control volume (CV), and a volume integral was performed for the convection, pressure, and shear stress term within the control volume. By utilizing the divergence theorem, the volume integrals were converted to the surface integrals over the boundary of the glottis, which were evaluated in the flow direction (y-direction) as follows:

(7) $${∭}_{CV}(\vec{V}\cdot \vec{\nabla })\vec{V}dV={∯}_{\partial V}\vec{V}(\vec{V}\cdot \vec{n}dS)=-\sum V_{1i}\cdot Q_{1i}+\sum V_{2i}\cdot Q_{2i},$$

(8) $${∭}_{CV}\vec{\nabla }PdV={∯}_{\partial V}P\vec{n}dS=\;-\sum P_{1i}\cdot A_{1i}+\sum P_{2i}\cdot A_{2i}+\sum P_{wi}\cdot A_{wi},$$

(9) $${∭}_{CV}\frac{1}{Re}{\vec{\nabla }}^{2}\vec{V}dV=\frac{1}{Re}{∯}_{\partial V}\left(\nabla V_{x},\;\nabla V_{y},\;\nabla V_{z}\right)\vec{n}dS=\frac{1}{Re}\left[\sum {\left(\frac{\partial V}{\partial x}dydz\right)}_{i}+\sum {\left(\frac{\partial V}{\partial z}dxdy\right)}_{i}\right],$$

where the subscripts 1, 2, and w denote the glottal inlet, outlet, and wall, respectively, and the subscript i(1,2,3,…) denotes the grid number through the corresponding area. Because the simulation results were not output at every time step, the volume integral of the unsteady acceleration term was obtained through balancing the momentum equation:

(10) $${∭}_{CV}\frac{\partial \vec{V}}{\partial t}dV=-{∭}_{CV}\left(\vec{V}\cdot \vec{\nabla }\right)\vec{V}dV-{∭}_{CV}\vec{\nabla }PdV+{∭}_{CV}\frac{1}{Re}{\vec{\nabla }}^{2}\vec{V}dV.$$

**Figure 7.**
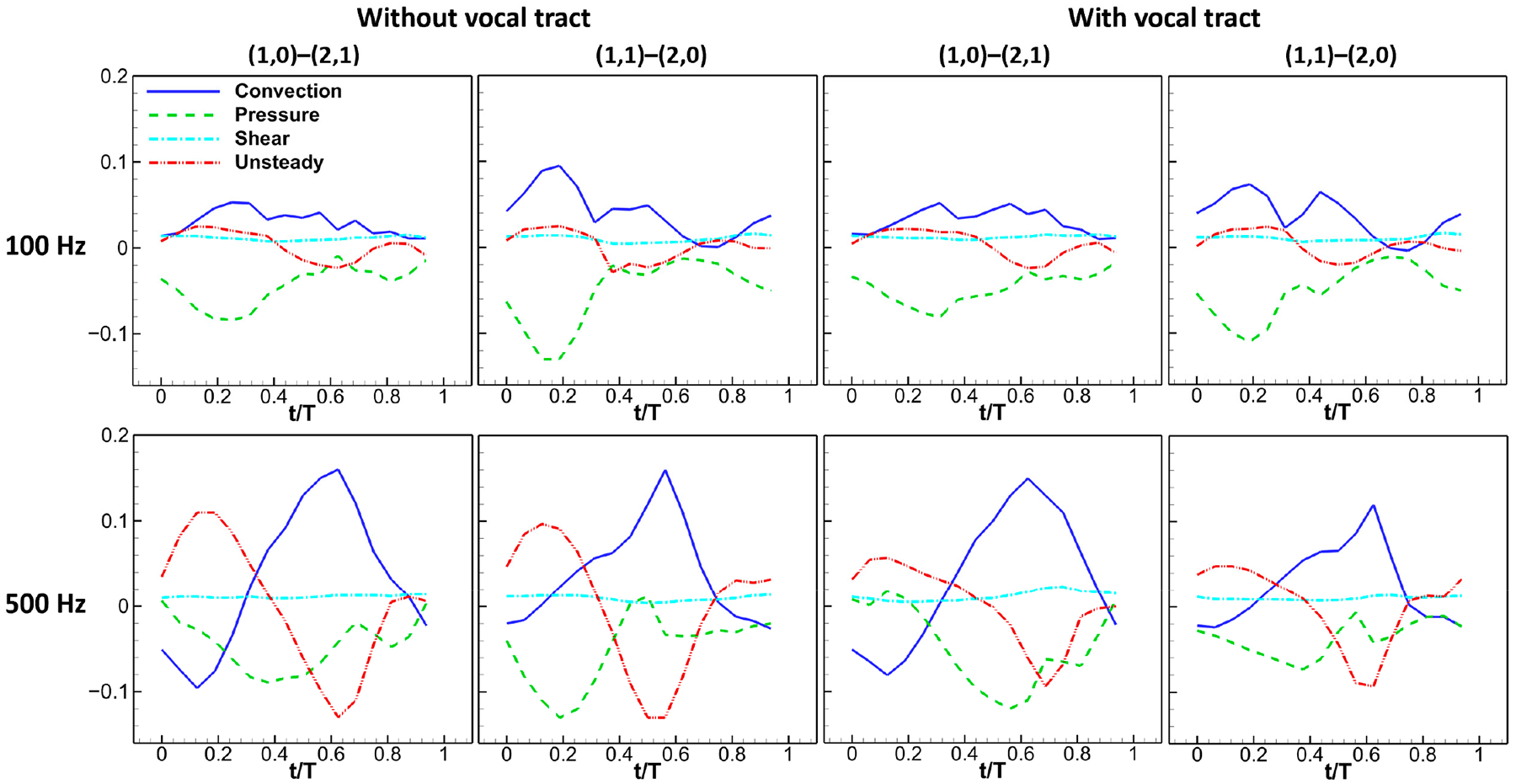
Variation in convection, pressure, shear, and unsteady term over one vibration cycle in each dynamic case.

Figure 7 depicts the variation in the convection, pressure, shear, and unsteady terms over a single vibration cycle in each dynamic case. At both 100 Hz and 500 Hz, the unsteady term exhibited a magnitude comparable to that of the convection and pressure terms. Throughout both the flow acceleration (unsteady term is positive) and deceleration (unsteady term is negative) stages, the magnitude of the unsteady term initially rose, reached a peak roughly halfway through the stage, and then declined. The term was nearly zero only briefly during the transition between flow acceleration and deceleration, as well as during early opening and near-closed phases. In most of the glottal cycle, the unsteady term was non-negligible at both frequencies. This observation contrasts with the previous finding that flow unsteadiness is only important during the short instances of flow initiation and shutoff but aligns with the results of Ringenberg et al. [21], who computed transglottal and dynamic pressures using the unsteady Bernoulli equation and found that the pressures were approximately only equal during a brief interval around the maximum glottal opening. By comparing the unsteady term curve with that of the flow rate error plot in Figure 4, it can be seen that the flow rate errors closely track the unsteady term variation, suggesting a strong association between the two.

Figure 7 also reveals that including a vocal tract has little impact on the magnitude of the unsteady term, as observed by comparing the cases with and without a vocal tract. However, as previously noted, a considerable increase in errors was observed in cases involving a vocal tract. This indicates that the increased errors are not caused by the unsteady effect of flow acceleration in the glottis but rather due to the inertance effect of the air column in the vocal tract, which causes the phase shift.

**Figure 8.**
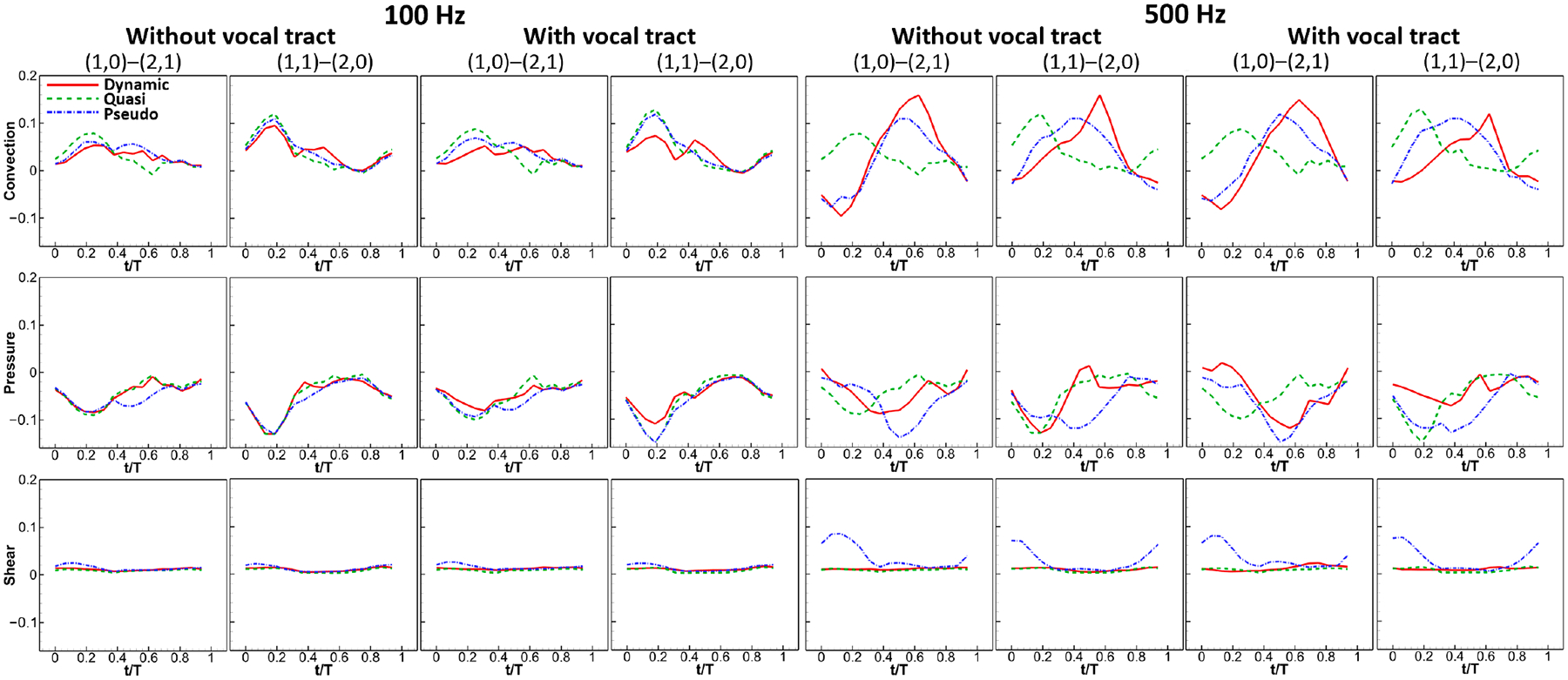
Comparison of convection, pressure, and shear term between the dynamic and quasi-steady/pseudo-static cases.

Figure 8 presents a comparison of the convection, pressure, and shear terms between the dynamic and quasi-steady/pseudo-static cases. At 100 Hz, the magnitudes and variations of the three terms were very similar among the three simulations, indicating that the dynamics of the glottal flow are comparable in the three simulations, and the contributions of unsteady acceleration are negligible. However, at 500 Hz, significant discrepancies in the convection, pressure, and shear terms were observed among the three simulations. Regarding the convection term, the quasi-steady predictions are substantially different compared to the dynamic simulations in both magnitude and variation. In contrast, the pseudo-static predictions closely resemble those of the dynamic simulations, and their magnitudes are comparable, suggesting that the displaced flow by wall motion could play a vital role in determining the convective acceleration. Compared with the dynamic simulations, the pressure term predicted by the two assumptions shows considerable differences in general, indicating large prediction errors in the transglottal pressure force and vocal fold drag at high frequencies. As for the shear term, the quasi-steady predictions were almost identical to the dynamic simulations, whereas the pseudo-static predictions were sometimes incorrect during the early opening and closed phases.

## 4. Discussion and Conclusions

This study aimed to assess the accuracy of the quasi-steady assumption in predicting glottal flow and pressure by analyzing errors in various glottal shapes and simulation setups, with and without the supraglottic tract, at vibration frequencies of 100 and 500 Hz. The findings indicate that the assumption yields minor errors at 100 Hz but substantial errors at 500 Hz. The momentum budget analysis suggests that the quasi-steady glottal flow closely resembles an unsteady glottal flow at 100 Hz but diverges significantly at 500 Hz, resulting in notable disparities in the momentum equation terms between quasi-steady/pseudo-static and dynamic simulations. Interestingly, our study reveals that unsteady acceleration is non-negligible even at low vibration frequencies, aligning with experimental results from Krane et al. [14], indicating that glottal flow is inherently unsteady. However, for the frequency range of normal speech phonation (~100–200 Hz), the unsteady acceleration contribution is small, and the quasi-steady approximation provides satisfactory predictions of glottal flow and pressure. Therefore, this assumption remains valid for such frequency ranges. As frequency increases, a point is reached where the predicted flow errors are no longer acceptable, and the quasi-steady assumption is no longer valid.

Similar to previous studies [9,10,12], our results indicate that errors in glottal flow are nonuniform throughout the vibration cycle. Previous findings have shown that errors are more pronounced during the early opening and late closing phases while remaining minimal during the middle interval of the cycle. This disparity in errors is believed to be related to the significance of glottal wall motion, as indicated by Krane and Wei [13]. During the initial and final stages of flow initiation and shutoff, the velocity of the glottal wall can be comparable to that of glottal flow, making the unsteady effects of wall motion noticeable. Conversely, during the stages around the maximum glottal opening, the wall velocity is much lower than the flow velocity, and the unsteady effects of wall motion are negligible. However, in our current study, we found that the maximum flow rate errors do not occur strictly during the stages of flow initiation and shutoff but rather sometime after the initiation point and before the closure point. This difference can be attributed to the fact that the two sets of glottal shapes never generate a fully closed glottis (as shown in Figure 2), meaning that the flow does not genuinely start from zero velocity and end with a complete stop. Nonetheless, our velocity ratio analysis $\left(v_{g}/v_{v}\right)$ still supports the previous argument. Generally, flow rate errors are more significant during the stages where the relative magnitude of airflow velocity and vocal fold velocity is small, while errors decrease as the relative magnitude increases.

This study has also shown that the inclusion of the inertance effect of the air column in the vocal tract can lead to a significant increase in the errors of quasi-steady flow at high frequencies. The momentum budget analysis indicates that including vocal tract inertance does not increase the magnitude of the unsteadiness of glottal flow, which suggests that increased errors are caused by the phase shift of the flow rate due to air inertance. According to lumped-element models of the vocal tract inertance by Titze [3] (Chapter 5), there exist nonlinear interactions between the vocal tract inertance and glottal airflow, skewing the flow rate waveform to the right and reducing the peak flow. These effects are also observed in the results of dynamic simulations in our study. However, such effects will not appear in the quasi-steady simulations since the flow is steady and there are no interactions between the glottal flow and air inertia in the vocal tract.

The two different sets of glottal shapes used in this study provide consistent results, suggesting the glottal shapes have little effect on the validity of the quasi-steady assumption. Compared to those in previous studies, the glottal geometries in the current study are much more complex, including the features of alternating convergent–divergent characteristics, left–right asymmetry, and longitudinal wave propagation, which could occur under irregular vocal fold vibrations. Despite the differences in glottal shape, the errors in the quasi-steady assumption were found to be similar to those reported in previous studies, indicating that glottal geometry has little effect on the validity of the assumption.

The quasi-steady and pseudo-static simulations yield very similar results at 100 Hz, indicating that the effect of glottal wall motion is negligible, and deviations from the dynamic simulations are primarily caused by local acceleration $\left(\frac{\partial \vec{V}}{\partial t}\right)$. As mentioned earlier, the importance of glottal wall motion depends on the ratio of wall velocity to flow velocity. At relatively low frequencies, the wall velocity is not significant except at the start and end of the flow cycle, making the unsteady effects of glottal wall motion negligible for most of the cycle. This finding is consistent with previous studies [13,19], which found that flow unsteadiness due to wall movement is not important. In Xi et al.’s [27] study on respiration, which involved a much lower frequency than phonation, the primary effect of glottal wall motion was on secondary flow in the transverse direction involving flow separation, swirling flows, and vortex shedding. The instability of the main flow in the streamwise direction was mostly influenced by the unsteady acceleration of the flow itself. While this study did not specifically examine phonation, the velocity ratio was similar to that in the current study, suggesting that the conclusion may still be applicable. However, further research is required to confirm this conclusion. In contrast, at high frequencies, such as 500 Hz, glottal wall motion can become important, as evidenced by the differences between the quasi-steady and pseudo-static simulations, particularly during the flow deceleration phase. Furthermore, the momentum budget analysis indicates that the displaced flow due to glottal wall motion may significantly contribute to the convective acceleration.

The results of this study suggest that there is no clear relationship between glottal flow unsteadiness and errors of MFDR. In the absence of a vocal tract, increasing vocal fold vibration frequency results in a larger phase shift between quasi-steady and dynamic simulations, but this does not appear to have a significant effect on the skewness of the flow rate waveform and, therefore, on the errors of MFDR. However, when the vocal tract is present, a clear difference in the skewness of the quasi-steady and dynamic flow rate waveforms is observed, resulting in more significant errors of MFDR. Nonetheless, there is still no dependence of MFDR errors on frequency. However, the spectral analysis in this study reveals that at high frequencies, the quasi-steady/pseudo-static prediction of H1–H2 has large errors, indicating that glottal flow unsteadiness still plays a role in determining vocal quality, which is perceptually important. Therefore, although the errors of MFDR do not depend on frequency, glottal flow unsteadiness remains a crucial factor in determining vocal quality at high frequencies.

One limitation of this study is that the shape of the supraglottic tract used in the simulation is not realistic. According to Saldías et al. [28], the real epilarynx airway is narrower, particularly around the vestibular fold area. This implies that the inertance effect of the vocal tract may be more significant if a more realistic supraglottic tract is taken into consideration. Another limitation is that only two sets of glottal shapes were studied due to the high computational cost of the simulation. Thus, the conclusion that the quasi-steady assumption is insensitive to glottal shapes is limited to the two sets of glottal shapes used in this study. To draw more general conclusions, future studies must investigate a wider range of glottal shapes, such as those involving higher modes in the combination or changing the prephonatory glottal configuration from uniform to divergent or convergent.

## Figures and Tables

**Table 1.** Overview of the previous studies on QSFA.

Study	VF Geometry	Frequency Investigated	Vocal Tract	Approach
[9]	Convergent	10~120 Hz	No	Experimental
[11]	Uniform, circular, and divergent	142 Hz	Yes	Theoretical and experimental
[10]	Convergent, straight, and divergent	70~120 Hz	Yes	Experimental
[19]	Straight, rounded, and Gaussian	45~315 Hz	No	Theoretical and experimental
[12]	Straight and rounded	38~540 Hz	No	Theoretical and experimental
[18]	Time-dependent	28.1~112.5 Hz	Yes	Experimental and numerical
[13]	Not applicable	100 Hz	Yes	Theoretical
[14]	Half cylinder	30~126 Hz	Yes	Theoretical and experimental
[20]	Reproduced from the coronal section and stained slices of the VFs	160 Hz	Yes	Experimental
[21,22]	Half cylinder	52.5~97.5 Hz	Yes	Theoretical and experimental
[23]	M5	112.5 Hz	No	Theoretical and experimental

**Table 2.** The value of the Strouhal number for each dynamic case.

	100 Hz		500 Hz	
	Without Vocal Tract	With Vocal Tract	Without Vocal Tract	With Vocal Tract
(1,0)–(2,1)	0.032	0.031	0.154	0.165
(1,1)–(2,0)	0.031	0.030	0.145	0.145

**Table 3.** Errors of important aerodynamic and sound spectrum parameters between quasi-steady/pseudo-static and dynamic cases. Phase shift is calculated as the phase difference in the peak flow between the quasi-steady/pseudo-static and dynamic cases. H1–H2 and H1–H4 are the spectral amplitude differences between the first two harmonics and the first and fourth harmonics, respectively. Spectral slope is determined using linear regression in MATLAB to fit the first 20 harmonics of each case.

		Without Vocal Tract		With Vocal Tract	
Frequency	Parameters	(1,0)–(2,1) Quasi/Pseudo	(1,1)–(2,0) Quasi/Pseudo	(1,0)–(2,1) Quasi/Pseudo	(1,1)–(2,0) Quasi/Pseudo
100 Hz	Peak flow (%)	−1.18/−2.18	−1.18/−3.28	−0.33/2.51	3.49/5.35
	Mean flow (%)	−2.24/−3.94	−3.36/−1.74	−0.61/−0.39	−2.14/2.61
	Phase shift (degree)	−11.34/−20.37	−8.82/−13.93	−25.48/−27.83	−24.89/−28.04
	MFDR (%)	0.49/−16.55	−9.51/−20.48	−13.83/−22.94	−13.19/−15.81
	H1–H2 (%)	−5.84/−9.23	−9.53/−0.20	−0.11/−7.65	−10.72/−1.31
	H1–H4 (%)	−7.19/−5.87	8.54/11.47	−17.07/−13.15	8.76/10.09
	Spectral slope (%)	12.30/14.75	27.64/44.44	8.75/13.00	14.78/6.05
500 Hz	Peak flow (%)	0.09/1.46	2.44/−6.79	28.12/32.95	37.71/29.57
	Mean flow (%)	−5.58/−10.26	−11.01/−8.56	14.07/10.68	4.46/10.75
	Phase shift (degree)	−21.27/−44.63	−27.62/−34.76	−59.11/−77.93	−61.89/−66.04
	MFDR (%)	0.79/−34.05	−2.18/−25.48	12.84/−16.15	17.69/−3.63
	H1–H2 (%)	−31.45/−43.76	−42.60/−21.55	−26.88/−41.37	−50.66/−32.46
	H1–H4 (%)	−21.90/−14.58	−19.46/−23.71	−2.43/8.23	−15.88/−19.58
	Spectral slope (%)	11.75/36.04	9.09/26.84	12.03/36.99	6.18/5.44

## Data Availability

All the data and materials in the manuscript are available upon request. The data are not publicly available due to privacy.
